# A chromosome-level genome assembly of the varied leaved jewelflower, *Streptanthus diversifolius,* reveals a recent whole genome duplication

**DOI:** 10.1093/g3journal/jkaf022

**Published:** 2025-03-18

**Authors:** John T Davis, Qionghou Li, Christopher J Grassa, Matthew W Davis, Sharon Y Strauss, Jennifer R Gremer, Loren H Rieseberg, Julin N Maloof

**Affiliations:** Department of Plant Biology, University of California, Davis, Davis, CA 95616, USA; National Key Laboratory of Crop Genetics and Germplasm Enhancement and Utilization, Sanya Institute of Nanjing Agricultural University, College of Horticulture, Nanjing Agricultural University, Nanjing, Jiangsu 210095, China; Department of Botany and Michael Smith Laboratories, University of British Columbia, Vancouver, British Columbia, Canada V6T 1Z4; Department of Organismic and Evolutionary Biology and the Harvard University Herbaria, Harvard University, Cambridge, MA 02138, USA; Department of Plant Sciences, University of California, Davis, Davis, CA 95616, USA; Department of Evolution and Ecology, University of California, Davis, Davis, CA 95616, USA; Department of Evolution and Ecology, University of California, Davis, Davis, CA 95616, USA; Center for Population Biology, University of California, Davis, Davis, CA 95616, USA; Botany Department, University of British Columbia, Vancouver, Canada V6T 1Z4; Department of Plant Biology, University of California, Davis, Davis, CA 95616, USA

**Keywords:** Brassicaceae, Hi-C, HiFi, *Streptanthus*, serpentine, thelypodieae, whole genome duplication

## Abstract

The Streptanthoid complex, a clade of primarily *Streptanthus* and *Caulanthus* species in the Thelypodieae (Brassicaceae) is an emerging model system for ecological and evolutionary studies. This complex spans the full range of the California Floristic Province including desert, foothill, and mountain environments. The ability of these related species to radiate into dramatically different environments makes them a desirable study subject for exploring how plant species expand their ranges and adapt to new environments over time. Ecological and evolutionary studies for this complex have revealed fascinating variation in serpentine soil adaptation, defense compounds, germination, flowering, and life history strategies. Until now a lack of publicly available genome assemblies has hindered the ability to relate these phenotypic observations to their underlying genetic and molecular mechanisms. To help remedy this situation, we present here a chromosome-level genome assembly and annotation of *Streptanthus diversifolius*, a member of the Streptanthoid Complex, developed using Illumina, Hi-C, and HiFi sequencing technologies. Construction of this assembly also provides further evidence to support the previously reported recent whole genome duplication unique to the Thelypodieae. This whole genome duplication may have provided individuals in the Streptanthoid Complex the genetic arsenal to rapidly radiate throughout the California Floristic Province and to occupy commonly inhospitable environments including serpentine soils.

## Introduction

Understanding the genetic basis of plant adaptation to diverse environments is the key to both understanding biodiversity and mitigating the impacts of climate change. Developing genetic and genomic resources for species or species complexes adapted to different environments enables research in this area. One attractive group of species for studying adaptation to different environments is the “Streptanthoid Complex”, a set of ca. 60 taxa belonging to the Thelypodieae (Burrell and Pepper 2006; Burrell 2010; Burrell *et al*. 2011). This complex comprises primarily *Streptanthus* and *Caulanthus*, has diverged over ca. 5 million years, and species diverged less than 2 million years generally have incomplete post-zygotic reproductive isolation (Christie and Strauss 2018). In spite of this relatively recent divergence time and genetic relatedness, species in the complex occupy a large range of environments including the deserts, mountains, and foothills of the California Floristic Province (CFP) (Howell 1957; Al-Shehbaz 2010; Cacho and Strauss 2014). The recent finding that different species within the complex vary in their sensitivity to seasonal rainfall timing and vulnerability to climate change associated shifts in rainfall patterns (Worthy *et al*. 2024) highlights the relevance of this complex in understanding environmental adaptation and the impacts of climate change.

In addition to its adaptation to different climates, *Streptanthus* is well known for extreme edaphic specialization, particularly to infertile serpentine soils. Serpentine is a unique soil known for its extraordinarily low Ca^2+^ to Mg^2+^ ratio, low levels of micronutrients, elevated heavy metal concentrations, and poor water retention (Brady *et al*. 2005; Kruckeberg 2006). Plants grow best when the Ca^2+^ to Mg^2+^ ratio of the soil is close to one or greater (Palm and Van Volkenburgh 2014) which makes the low Ca^2+^ to Mg^2+^ ratio of particular interest as Mg^2+^ concentrations in serpentine soils can exceed those of Ca^2+^ by almost tenfold (Alexander 2007). Serpentine soil usage has 4 or 5 independent origins in the Streptanthoid Complex (Cacho and Strauss 2014) and more than half of the ∼35 known species in the complex are endemic to serpentine soils (Safford *et al*. 2005; Al-Shehbaz 2010; Baldwin *et al*. 2012). The Complex has been the subject of several classic studies on the evolution of edaphic specialization and is an emerging model system for understanding the evolution of edaphic specialization in herbaceous plants. *Streptanthus* has also been the subject of molecular phylogenetic investigations, providing a robust phylogenetic context in which to examine the evolution of specific traits during diversification (Cacho and Strauss 2014; Weber *et al*. 2018; Cacho *et al*. 2021).

To increase and improve genomic resources for research in *Streptanthus* and its allies, we carried out whole-genome shotgun sequencing, Hi-C sequencing, and PacBio HiFi sequencing on the nuclear genome of the varied leaved jewelflower, *S. diversifolius.* While *S. diversifolius* is not found on serpentine soils, it has the smallest genome known in the genus (0.36 GB), and is a known diploid (2*n* = 28) (Cacho *et al*. 2021). A reference genome for *S. diversifolius* will facilitate gene discovery, genetic mapping, and re-sequencing of other *Streptanthus* species, all in support of ongoing studies. The reference genome sequence described here will allow mechanistic knowledge from the related model genera *Arabidopsis* and *Brassica* to be leveraged for understanding the molecular basis of serpentine specialization, heavy metal tolerance and hyperaccumulation, and climate adaptation. Such insights may lead to improvements of closely related crops in the Brassicaceae family, as well as industrial applications in phytoremediation and phytomining. Additionally, a greater understanding of the Streptanthoid complex will allow us to build upon previously work including the evolution of glucosinolate defense (Cacho *et al*. 2015) and the seasonal germination niche (Gremer *et al*. 2020; Worthy *et al*. 2024). Combined these analyses will assist in the conservation and management of these species, many of whom are currently endangered or threatened.

## Methods and materials

### Plant collection, DNA isolation, and sequencing

Plant collection, DNA isolation, and sequencing occurred in 3 different rounds. In the first round, several whole, flowering plants of *S. diversifolius* were collected in April 2013 from a naturally occurring population on Table Mountain, Butte County, California (D. O. Burge 1389; voucher deposited at DAV). Tissues of these plants were dried on a silica gel desiccant at room temperature before being returned to the lab at the University of British Columbia. Total genomic DNA was extracted from the leaves, stems, and unopened flower buds of a single plant using the Qiagen (Limburg, Netherlands) DNeasy Kit according to the manufacturer's instructions. A total of 24 extractions were performed. Pooled extractions were then concentrated by adding 1/10 volume of 3-M sodium acetate, pH 5.2, and 2 volumes of −20°C 95% EtOH. After centrifugation, the DNA pellet was dried and resuspended in pure, nuclease-free water. Aliquots of the same DNA preparation were then used to construct 4 sequencing libraries according to the manufacturer's protocols (Illumina, http://www.illumina.com) at the Innovation Centre, Genome Quebec. Library insert sizes were chosen to be compatible with the Allpaths-LG genome assembler (Gnerre *et al*. 2011). We prepared 2 libraries designed to overlap when sequenced as paired ends: a 180 bp library sequenced on an Illumina HiSeq with 2 × 100 bp reads, and a 450 bp library sequenced on an Illumina MiSeq with 2 × 300 bp reads. We also prepared 2 mate-pair libraries with insert sized of approximately 4,700 and 8,200 bp.

In the second round, seeds from *S. diversifolius* were collected in 2016 from a naturally occurring population on Table Mountain, Butte County, California. It should be noted that this is a separate seed collection from that described above. Eight cones containing a mixture of 50% sand and 50% Ron's Mix (a UC Davis custom mix consisting of 1 part coarse sand, 1 part compost (redwood shavings and turkey manure), 1 part peat moss, and 3 pounds/yard Dolomite) were saturated with nutrient water (N:P:K 2:1:2). A small divot was then made in each cone where 3–4 seeds were placed before being covered with a small amount of the soil mixture. The cones were then placed in a rack and covered with plastic wrap to prevent the top layer of the soil from drying out. The covered rack was then placed in a growth chamber set to 22˚C with a light/dark cycle of 12/12. Two weeks after being placed in the growth chamber, the plastic wrap was removed and the recently germinated seedlings were exposed to the air. The seedlings remained in the growth chamber with nutrient water being provided every other day. Once the seedlings on average had attained approximately 8–10 true leaves, 2 plants were randomly selected for Hi-C sequencing. Young leaves from each plant were collected separately until approximately 0.5 g were obtained. The leaves from each plant were than processed separately using Phase Genomics' Proximo Hi-C Kit version 3.0 following the standard plant protocol. The libraries where then sent to the University of California, Davis Genome Center where 150 bp paired-end sequencing was performed using an Illumina NovaSeq 6000. A total of ∼240 million read pairs were sequenced resulting in ∼200 × coverage of the genome.

In the third round, leaf tissue from 3 different *S. diversifolius* individuals located at Table Mountain, Butte Country, CA were harvested individually in May 2023. The 3 samples weighing 0.66, 0.59, and 0.52 g were placed on ice and transported to UC Davis where they were flash frozen in liquid nitrogen upon arrival. All 3 samples were then sent to the UC Davis genome center for HMW DNA extraction. DNA was successfully extracted from the 0.66 g of sample and used for sequencing and the remaining 2 leaf samples were kept for backup. Sequencing was performed at the UC Davis genome center on PacBio's Revio sequencing platform. The reads were then processed using SMRT Link version 12.0.0.177059 resulting in 4,689,053 HiFi reads with a mean read length of 11,052 base pairs and mean read quality of 30. The HiFi yield of 51.8 Gb represents a genome coverage of ∼144 ×.

### Genome assembly

Starting with the Illumina short reads from round one, raw sequence reads were filtered and trimmed of their adapter sequences using Trimmomatic (Bolger *et al*. 2014). Trimmed and filtered reads were assembled using Allpaths-LG with the haploidify = T parameter. Biological contaminants in the resulting assembly were identified against the NCBI NT database using blastn megablast (Sayers *et al*. 2022). Seven scaffolds with a match of at least 85% and at least 300 bp to a database sequence belonging to non-plant taxa were removed from the Allpaths-LG assembly. Artificial contaminants were identified using NCBI's VecScreen protocol. Contaminants at scaffold ends were removed; artifacts within scaffolds were masked.

To improve the contiguity of the assembly from round one, the Hi-C sequencing data from round 2 was added to the assembly. The Hi-C reads were trimmed using Trimmomatic version 0.39 in paired-end mode with the parameters ILLUMINACLIP:adapters.fa:2:30:10 LEADING:3 TRAILING:3 SLIDINGWINDOW:4:15 MINLEN:36 resulting in ∼180 million surviving read pairs. The reads were then mapped to round one scaffold using the Arima-Hi-C Mapping Pipeline (https://github.com/ArimaGenomics/mapping_pipeline). Following mapping, the alignment file was sorted by read name using Samtools (Li *et al*. 2009) sort. The sorted alignment file along with the scaffolds were input into the Hi-C scaffolding program YaHS (Zhou *et al*. 2023).

The Hi-C scaffolded assembly showed signs of a possible whole genome duplication along with highly probable mis-joins of the contigs. To investigate these 2 occurrences, a new genome assembly was created on the Jetstream2 cloud computational platform (Hancock *et al*. 2021) using the HiFi sequencing data from round three. The HiFi reads were converted from BAM format to FASTQ format for assembly. The FASTQ reads were assembled using the program HiFiasm version 0.19.5-r593 (Cheng *et al*. 2021) using default parameters with the –primary flag selected.

A telomere analysis (methods below) of the HiFiasm generated contigs revealed that 11 of these contigs captured telomeres at both ends of the contig, while an additional 6 contigs over 1 Mb in length each had telomeric sequences on a single end. To attempt to join these contigs and create a telomere-to-telomere genome assembly, the Hi-C sequencing reads were employed to scaffold these 6 sequences. The Hi-C raw sequencing reads generated in round 2 were trimmed with Trimmomatic (Bolger *et al*. 2014) version 0.39 in paired-end mode with SLIDINGWINDOW:4:15. The reads were mapped to all HiFiasm generated contigs using the short read mapping preset in Minimap2 (Li 2018, 2021) version 2.17-r941. The resulting BAM files were sorted, updated with mate coordinates, duplicates marked, and indexed using Samtools (Li *et al*. 2009). From these BAM files, the alignments of the Hi-C reads to the 6 contigs of interest were extracted with Samtools.

The 6 contigs were then scaffolded using Hi-C scaffolding program YaHS, with a minimum mapping quality of 20, no contig error correction, and no scaffold error correction to minimize contigs being broken into smaller sequences and reordered. To visually assess the results of scaffolding on the 6 contigs, the resulting scaffolded contigs were first processed with Juicer and Juicer Tools (Durand *et al*. 2016) Juicebox was then used to visually assess scaffolding with interactive Hi-C contact maps. It was determined that the 6 contigs had been scaffolded appropriately into 3 scaffolds and that no manual curation was necessary. To confirm proper scaffolding, telomeric sequences were examined, and the 3 scaffolds were shown to have telomeres on both terminal ends of each scaffold.

To correct for contig breaking and reordering introduced by Hi-C scaffolding, the 6 contigs of interest were scaffolded back to the Hi-C scaffolds with the scaffolding program in RagTag (Alonge *et al*. 2022) version 2.1.0. This re-scaffolding resulted in 3 scaffolded sequences, and telomeres were again identified at both terminal ends of each scaffold. Final assemblies were generated by selecting the 11 telomere-to-telomere contigs generated by HiFiasm and the 3 scaffolds generated after re-scaffolding with RagTag. This final telomere-to-telomere assembly was used for the rest of the analysis.

### Repeat and gene annotation

The HiFi assembly was annotated using the MAKER pipeline (Cantarel *et al*. 2008; Holt and Yandell 2011). First a custom repeat library for *S. diversifolius* was made using the Maker-P pipeline (Campbell *et al*. 2014a, 2014b). Augustus (Keller *et al*. 2011) retraining parameters were also calculated using BUSCO v4.1.4 (Manni *et al*. 2021) in long and genome mode and the brassicales_odb10 lineage dataset. The assembly was input into Maker v3.01.04 along with the repeat library, Augustus retraining parameters, and transcripts from 6 other *Streptanthus* clade species comprised of *Caulanthus anceps*, *Caulanthus amplexicaulis*, *Caulanthus inflatus*, *Streptanthus breweri*, *Streptanthus glandulosus*, and *Streptanthus tortuosus* (https://doi.org/10.5061/dryad.t1g1jwt99) to strengthen prediction power (Supplementary Table 1 in file Supplemental Material). A second round of Maker was performed using the same inputs except this time a newly made *S. diversifolius* hmm file made using Snap (Korf 2004) v2006-07-28 and the GFF file created in the first round of Maker were included. In the second round of Maker the parameter of AED = 0.5 was also set, previously AED = 1, to remove transcripts which had weak evidence support. A total of 40,605 gene models were created. These gene models were post processed following the MAKER Tutorial for WGS Assembly and Annotation Winter School 2018 (https://weatherby.genetics.utah.edu/MAKER/wiki/index.php/MAKER_Tutorial_for_WGS_Assembly_and_Annotation_Winter_School_2018) where they were aligned to the UniProt database using blastp and processed using interproscan (Jones *et al*. 2014). These results were then incorporated into the final annotations (Supplementary Tables 2 and 3 in Supplementary Material).

Genes and gene features were lifted over from the gff file generated for the HiFiasm contigs to the telomere-to-telomere assembly using Liftoff version 1.6.3 (Shumate and Salzberg 2021). Features were mapped if they aligned with coverage ≥ 0.99 and child features aligned with sequence identity ≥ 0.99.

### Telomere annotation

To test whether we had captured telomeres, we used the Biostrings package (Pagès *et al*. 2024) to count occurrences of the 21 bp sequence “TTTAGGGTTTAGGGTTTAGGG” and its reverse complement (representing 3 repeats of the canonical plant telomere repeat sequence) in 1,000,000 base windows across the genome. We retained windows that had at least 20 copies of the query sequence.

### Genomic data sources

To investigate the possibility of a whole genome duplication, comparative genomic analyses were performed. The genome data and annotation files of following Brassicaceae species were obtained from public repositories: *Caulanthus amplexicaulis* v1.1, *Eutrema salsugineum* v1.0 (Yang *et al*. 2013), *Schrenkiella parvula* v2.2 (Dassanayake *et al*. 2011), *Stanleya pinnata* v1.1, *Arabidopsis thaliana* Araport 11 (Cheng *et al*. 2017), *Arabidopsis lyrata* (Hu *et al*. 2011) from Phytozome (https://phytozome-next.jgi.doe.gov/) (Goodstein *et al*. 2012); *Cardamine hirsute* (Gan *et al*. 2016) from *Cardamine hirsuta* v1.0 (http://chi.mpipz.mpg.de/index.html); *Euclidium syriacum* v1.1 was from NCBI accession number (GCA_900116095.1); *Draba nivalis* v1.0 (Nowak *et al*. 2021) from Dryad (https://datadryad.org); *Arabis alpina* v5.1 (Jiao *et al*. 2017) from (http://www.arabis-alpina.org/index.html); *Thlaspi arvense* genome (Geng *et al*. 2021) from (http://pennycress.umn.edu/); *Brassica oleracea* v1.0 from Genoscope (http://www.genoscope.cns.fr/externe/plants/chromosomes.html); *Raphanus raphanistrum* ssp. *raphanistrum* v1.0 (Zhang *et al*. 2021) from (https://ngdc.cncb.ac.cn/gwh/#) under accession number PRJCA003033; and *Aethionema arabicum* v3.0 (Fernandez-Pozo et al., 2021) from (https://plantcode.cup.uni-freiburg.de/aetar_db/downloads.php).

### Alignment to other genomes

To assess the contiguity of the Hi-C and HiFi draft assemblies, the *S. diversifolius* genomes were aligned to a reference *A. thaliana* genome (TAIR 10; Berardini *et al*. 2015). Two analyses were done to facilitate different visualizations. For a dotplot visualization, the genomes were aligned in protein space using the promer alignment tool contained within the MUMmer software package (Marçais *et al*. 2018). Promer was run using the ‘maxmatch’ flag and the default setting for the other parameters. Following alignment, the delta files were then filtered using delta-filter with minimum alignment length and sequence identity cutoffs of 1,000 bp and 85% respectively. The filtered delta files were then plotted using mummerplot. For an alluvial plot visualization, the genomes were aligned in protein space and plotted using MCscan (Tang *et al*. 2008) from the JCVI toolkit (Tang *et al*. 2024), with a minimum span of 30 genes and otherwise default settings. For both plots, syntenic regions were colored based on the ancestral crucifer karyotype (ACK) blocks (Lysak *et al*. 2016) as defined for *A. thaliana.* Although *S. diversifolius* and *A. thaliana* belong to different lineages within the *Brassicaceae* family, we chose *A. thaliana* for this comparison because of its chromosome-level assembly, lack of recent whole genome duplications, and well-defined ACK blocks. To compare *S. diversifolius* to more closely related species, we also used MCscan for synteny comparisons with *Schrenkiella parvula* v2.2 (Dassanayake *et al*. 2011) *Eutrema salsugineum* v1.0 (Yang *et al*. 2013), and *Caulanthus amplexicaulis* v1.1.

### Species tree construction

To construct a species tree, we identified single-copy orthogroups from the proteins of the 14 genomes listed above, including *S. diversifolius*, using OrthoFinder (Emms and Kelly 2019). This identified 118 single-copy orthogroups that were then aligned using MAFFT (Katoh and Standley 2013). Pal2nal was used to perform codon alignment (Suyama *et al*. 2006). TrimAl was used to trim poorly aligned segments from the alignments (Capella-Gutiérrez *et al*. 2009). Based on high-quality alignments, the phylogenetic trees for the 118 single-copy orthogroups were constructed using the IQ-TREE (Minh *et al*. 2020). Finally, the species tree was inferred using ASTRAL-III (Zhang *et al*. 2018).

### Identification of whole genome duplication

To identify whole-genome duplication events in *Streptanthus*, we compared the *S. diversifolius* genome with 13 other genomes (all species listed above except *E. salsugineum*). Initially, the DupGene_Finder pipeline (Qiao *et al*. 2019) was used to identify WGD genes across the 14 genomes, including *S. diversifolius*, while removing tandem genes. Subsequently, the Ka_Ks_pipeline (Qiao *et al*. 2019) was utilized to calculate the number of substitutions per synonymous site (Ks) values for WGD pairs. We made some modifications to the previous Ks fitting process (identify_Ks_peaks_by_fitting_GMM) to allow fitting based on the Ks values of gene pairs. To place the WGD events, based on the phylogenetic relationships of the 15 species, we identified orthologs between *S. diversifolius* and *C. amplexicaulis* and orthologs between *S. diversifolius* and *S. pinnata*, calculated the Ks values for these orthologs, and plotted the Ks distribution. The common ancestor of Brassicaceae underwent an α-WGD event approximately ∼35 Mya (Qiao *et al*. 2019), corresponding to a Ks value of 0.85 for Arabidopsis in our study. Therefore, we estimated that the recent WGD of Streptanthus occurred approximately 7.38 Mya (Ks value 0.18).

### Sub-genome differentiation

Two methods were used to look for possible differentiation among sub-genomes. First, SubPhaser (Jia *et al*. 2022) was run with default parameters (*k* = 15, *q* = 200) to look for evidence of unique kmers among homeologs. Second, the CoGe framework (Lyons and Freeling 2008) was used to align *S. diversifolius* coding regions against those from *Arabidopsis thaliana* and to perform a fractionation bias analysis (Joyce *et al*. 2017). A syntenic ratio of 2:1 *S. diversifolius*: *A. thaliana* was specified for Quota Align (Tang *et al*. 2011); otherwise, default parameters were used.

### Gene family evolution

To identify expanded and contracted gene families, the CAFE5 (Mendes *et al*. 2021) software was utilized. Following the CAFE5 documentation, orthogroups previously identified by OrthoFinder with more than 100 gene copies in one or more species were excluded in downstream analysis. The previously described species tree was transformed into an ultrametric tree, using MCMCtree which is incorporated in PAML v4.10.7 (Yang 2007), utilizing the age of the most recent common ancestor of *A. arabaicum* and the remaining 13 other species as 35.2 million years ago (MYA), based on a previous study (Nowak *et al*. 2021). The ultrametric tree and the filtered orthogroups were then used as input for CAFE5. Testing was performed on different –k parameters to determine the optimal K value based on the Model Gamma Final Likelihood value, where the best –k value was found to be 2. Finally, significantly expanded and contracted families were extracted from the result files.

### Homoeolog retention and loss analysis

To identify genes that were retained as duplicates or that reverted to single copy after the WGD, GENESPACE (Lovell *et al*. 2022) was used to identify syntenic “pangenes” among *E. salsigineum, S. parvula, S. diversifolius,* and *C. amplexicaulis*, including homoeologs among the latter 2 species that include the WGD. We filtered the 21,343 pangenes to keep those that were single copy in the ancestral group of *E. saligineum* and *S.parvula* and either single or double copy in the other 2species. Of the 16,490 pangenes passing this filter, 10,492 were retained as duplicates in both *S. diversifolius* and *C. amplexicaulis,* 1,834 reverted to single copy in both species, 709 were retained as duplicates only in *S. diversifolius,* and 3,455 were retained as duplicates only *C. amplexicaulis.* Next, we used the R/Bioconductor package topGO (Alexa and Rahnenfuhrer 2024) to search for over-represented GO terms among these different categories of genes. We used the “weight01” algorithm, which blends the “elim” and “weight” algorithms to account for the hierarchical topology of GO categories (Alexa *et al*. 2006). *P*-values were not corrected for multiple testing, following recommendations in the topGO user manual for the “weight” and “elim” algorithms.

### General data analysis

Base R (R Core Team 2024) and the tidyverse suite of libraries (Wickham *et al*. 2019) were used extensively.

### Github repository

The scripts used for the analyses described in this manuscript are available at https://github.com/MaloofLab/Davis_Dimensions_Sdiv_Assembly

## Results

The construction of this new *S. diversifolius* whole genome assembly took place over multiple rounds, with each round producing an improved assembly compared to its predecessor. Starting with an Illumina short read assembly and progressing to a HiFi assembly, the results of each iteration are described below. Following the assembly are the results of analyses investigating the presence of a suspected whole genome duplication.

### Round 1: Illumina assembly

The 4 Illumina sequencing libraries, HiSeq with 2 × 100 bp reads, MiSeq with 2 × 300 bp reads, and mate-pair libraries with insert sizes of approximately 4,700 and 8,200 bp were used for the initial assembly. Assembly was performed using the whole-genome shotgun assembler ALLPATHS-LG and subsequently postprocessed using NCBI's VecScreen protocol. The resulting assembly had a total length of 314 Mb, a scaffold N50 of 470 Kb, and comprises 4,627 scaffolds (Table 1). Genome completeness of the assembly using BUSCO version 5.5.0 and the embryophyta odb10 database found the assembly to have a complete BUSCO percentage of 98.7% with an approximate even split between single-copy and duplicate BUSCOs (Table 2).

**Table 1.** Assembly statistics for each round of assembly.

Assembly	Number of Scaffolds	Number of Contigs	Total Length (bp)	Gaps (bp)	Scaffold N50	Contigs N50
Round 1	4,627	20,342	314,016,962	35,044,678	470 Kb	49 Kb
Round 2 Full	3,920	20,571	314,205,162	35,232,878	21 Mb	49 Kb
Round 2 1 Mb	17	13,149	280,150,441	25,082,127	22 Mb	55 Kb
Round 3 Full	1,183	1,183	402,145,164	0	23.5 Mb	23.5 Mb
Round 3 1 Mb	18	18	345,697,404	0	24 Mb	24 Mb
Round 3 T2T Full	1180	1180	402,145,464	300	24 Mb	24 Mb
Round 3 T2T Chr	14	14	336,183,238	300	24 Mb	24 Mb
Arabidopsis (TAIR10)	7	99	119,668,634	186,207	23 Mb	11 Mb

**Table 2.** BUSCO summary statistics. Analysis completed using BUSCO version 5.5.0 in genome mode and the embryophyta odb10 dataset.

Assembly	Complete %	Single-Copy %	Duplicated %	Fragmented %	Missing %	# BUSCOS
Stage 1	98.7	50.6	48.1	0.7	0.6	1614
Stage 2 Full	98.8	50.8	48	0.7	0.5	1614
Stage 2 1 Mb	98	51	47	0.7	1.3	1614
Stage 3 Full	99.6	47.1	52.5	0.1	0.3	1614
Stage 3 1 Mb	99.6	47.1	52.5	0.1	0.3	1614
Round 3 T2T Full	99.6	47.1	52.5	0.1	0.3	1614
Round 3 T2T Chr	99.6	47.6	52.0	0.1	0.3	1614
Arabidopsis (TAIR10)	99.3	98.6	0.7	0.2	0.5	1614

### Round 2: Hi-C assembly improves contiguity and suggests possible whole genome duplication

Looking to improve the contiguity of the Illumina assembly, 2 Hi-C sequencing libraries were prepared using tissue from 2 separate *S. diversifolius* plants whose seeds came from a population in the same geographic location as the original plant samples. Both sequencing libraries were aligned to Illumina assembly using the Aria Hi-C Mapping Pipeline and the resulting alignment files were input into the Hi-C scaffolding program YaHS. The scaffolded assembly showed signs of improvement compared to the original Illumina assembly. The scaffolding resulted in the overall size of the assembly increasing slightly due to the introduction of scaffolding gaps. The main improvements were a decrease in the number of scaffolds from 4,627 to 3,920 scaffolds and an increase in the scaffold N50 from 470 Kb to 21 Mb. Additionally, 17 scaffolds were greater than 1 Mb in length and encompassed 89% of the total assembly length (Table 1). Scaffolding did not have a significant effect on BUSCO composition and the majority of the BUSCOs were found to be contained in the 17 previously mentioned scaffolds (Table 2).

To examine the quality and contiguity of the scaffolded assembly, the assembly was aligned to a reference *A. thaliana* genome in protein space using promer from the MUMmer bioinformatic tool suite. The resulting plot displayed a mirroring pattern whereby scaffolds had apparent end-to-end joining of duplicate chromosomes (Supplementary Fig. 1 in file Supplemental Material). This mirroring pattern suggested the possibility of 2 different phenomena occurring. The first was a potential whole genome duplication due to the frequency and size of the mirrored regions and the second was the presence of mis-joins in the assembly given the proximity of the mirrored regions to their counterparts. While the latter was deemed to be most likely a scaffolding artifact, the former was found to be biologically plausible given the frequency of recent whole genome duplications found across the different tribes of the Brassicaceae family (Kagale *et al*. 2014; Mandáková *et al*. 2017).

### Round 3: HiFi further improves assembly and removes artifactual joins

**Fig. 1.**
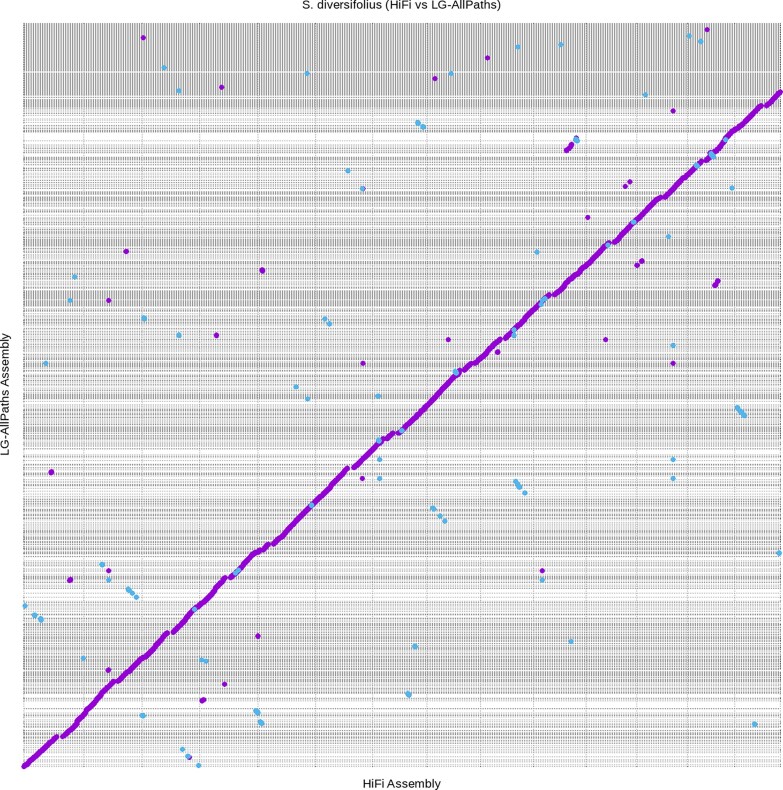
The 14 *S. diversifolius* chromosomes from the HiFiasm assembly aligned to all scaffolds in the LG-AllPaths assembly. Alignment was performed using nucmer and plotted using mummerplot, both programs included in the MUMmer bioinformatic toolkit. Purple lines indicate regions of alignment in the forward direction and blue lines indicate regions of alignment in the reverse direction. Gray dashed lines indicate separate scaffold in each assembly.

To further investigate the possibility of a whole genome duplication and correct mis-joins created in the Hi-C scaffolding process, a third round of sequencing was performed. Leaf tissue was collected from individuals located in the same geographic region as those of the first 2 rounds and extracted high molecular weight DNA was prepped and sequenced on the PacBio Revio sequencing platform. Following postprocessing, the HiFi reads were assembled using the long read assembler HiFiasm. The 11 largest contigs from HiFiasm had telomeres on both ends and 6 of the remaining 7 contigs larger than 1 MB had telomeres on one end. We used Hi-C reads from round 2 to scaffold the 6 contigs with single telomere ends, yielding a final assembly with the expected number of 14 telomere-to-telomere chromosomes (Supplementary Fig. 2 in file Supplemental Material). The HiFi assembly was an improvement over the assemblies created in both rounds one and two. Total sequence length increased to 402 Mb with 83.6% of the total sequence length contained in the 14 chromosomes. Along with the increase in assembly size, the total number of scaffolds dropped to 1,180 and the scaffold N50 increased to 24 Mb (Table 1). The HiFi assembly also saw an improvement in complete BUSCOs and now showed slightly more duplicated than single-copy BUSCOs. All BUSCOs were also contained within the previously mentioned 14 chromosomes (Table 2). When compared to the round one assembly, most of the sequence is shared between the 2 assemblies, with the main difference being the contiguity of scaffolds (Fig. 1).

Annotation was completed using the Maker-P pipeline. Maker analysis of the HiFi telomere-to-telomere assembly from round 3 predicted 40,605 protein-coding genes after filtering (Supplementary Table 2 in file Supplemental Material). Of these 40,605 protein-coding genes, 34,644 are found on the 14 telomere-to-telomere chromosomes.

### Comparison of rounds 2 and 3

**Fig. 2.**
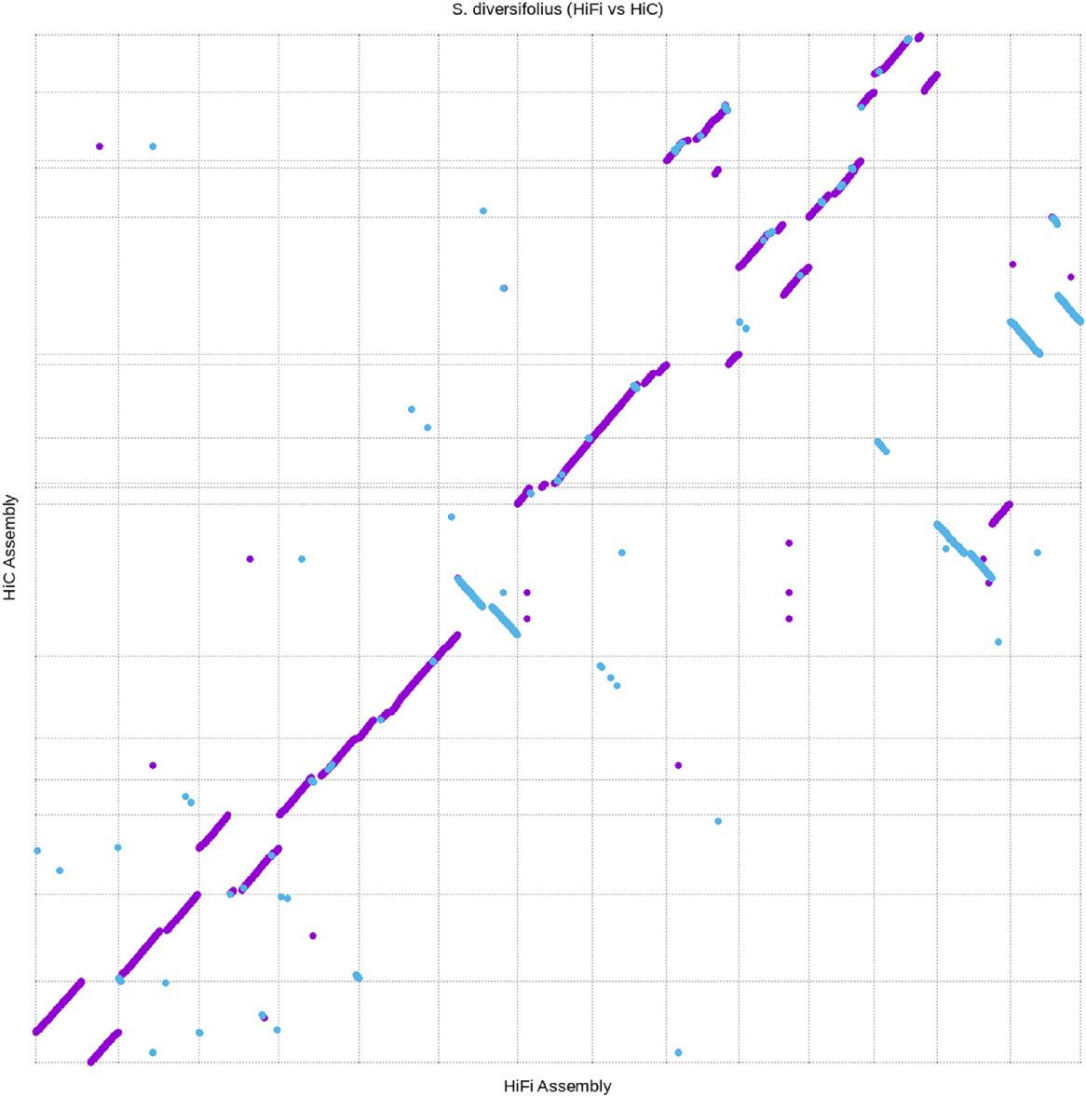
The 14 chromosomes from the HiFiasm assembly aligned to all scaffolds greater than 1 Mb in the Hi-C assembly. Alignment was performed using nucmer and plotted using mummerplot, both programs included in the MUMmer bioinformatic toolkit. Purple lines indicate regions of alignment in the forward direction and blue lines indicate regions of alignment in the reverse direction. Gray dashed lines indicate separate scaffold in each assembly.

**Fig. 3.**
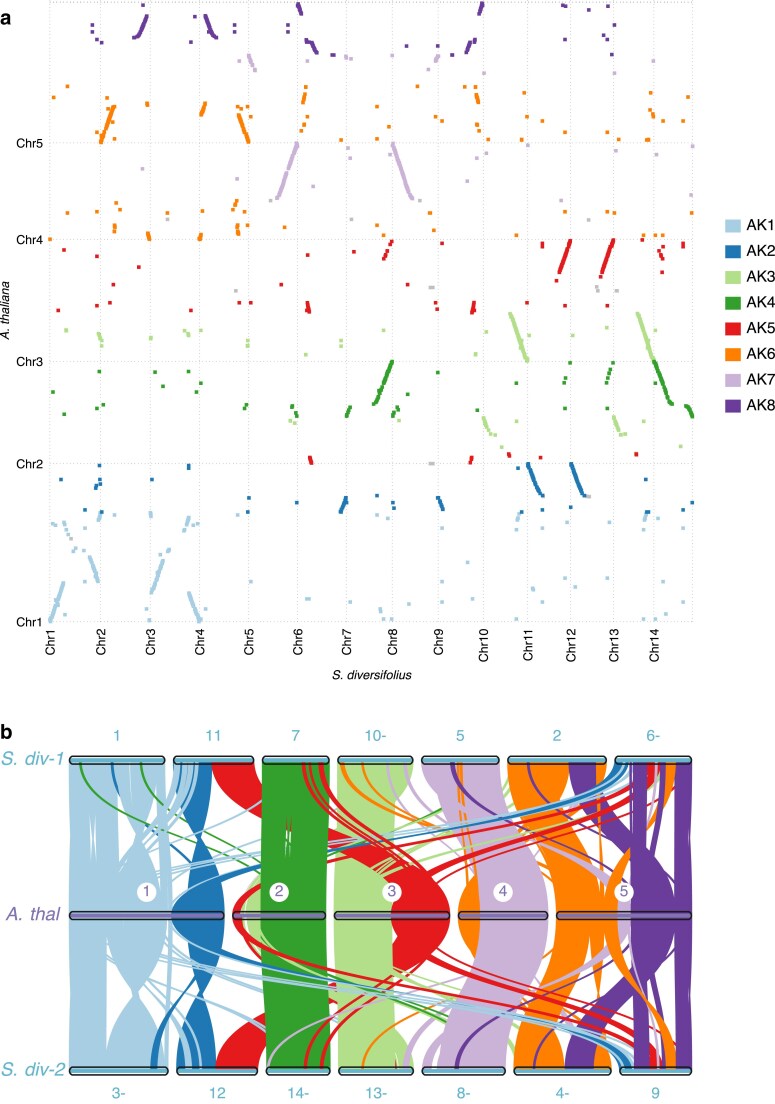
Synteny comparison with *Arabidopsis thaliana*. The 14 chromosomes from the HiFiasm assembly aligned to the TAIR 10 A. thaliana genome assembly. a) Dotplot showing similarity between A*. thaliana* and *S. diversifolius*. Alignment was performed using Promer and plotted using mummerplot. Colors correspond to the 8 ancestral crucifer karyotype (ACK) blocks as defined in *A. thaliana*, with the exception of grey which corresponds to regions which do not belong to an ACK block. ACK block boundaries are based on gene locations summarized in Lysak et. al 2016. b) Synteny plot produced with JVCI MCscan (Tang *et al*. 2008, 2024). The *S. diversifolius* homoeologous chromosomes were separated into 2 groups (shown in the top and bottom rows) to better illustrate the duplication. A “–” after a scaffold number indicates that it is plotted in reverse orientation for better visualization. Colors are ACK blocks, as in A.

The HiFi assembly was created to correct mis-joins created in the round 2 Hi-C scaffolding process and validate the existence of a recent whole genome duplication. When aligned against one another, the round 2 Hi-C scaffolded and HiFi assembly were mostly congruent. (Figure 2). The HiFi assembly was aligned to *A. thaliana* in the same way as the Hi-C scaffolded assembly. The mirroring pattern observed in the Hi-C scaffolded assembly was no longer present. Evidence of a recent whole genome duplication was present as seen by the presence of duplicate ancestral genomic regions spread throughout the genome (Fig. 3a).

### Whole genome duplication timing and mechanism

**Fig. 4.**
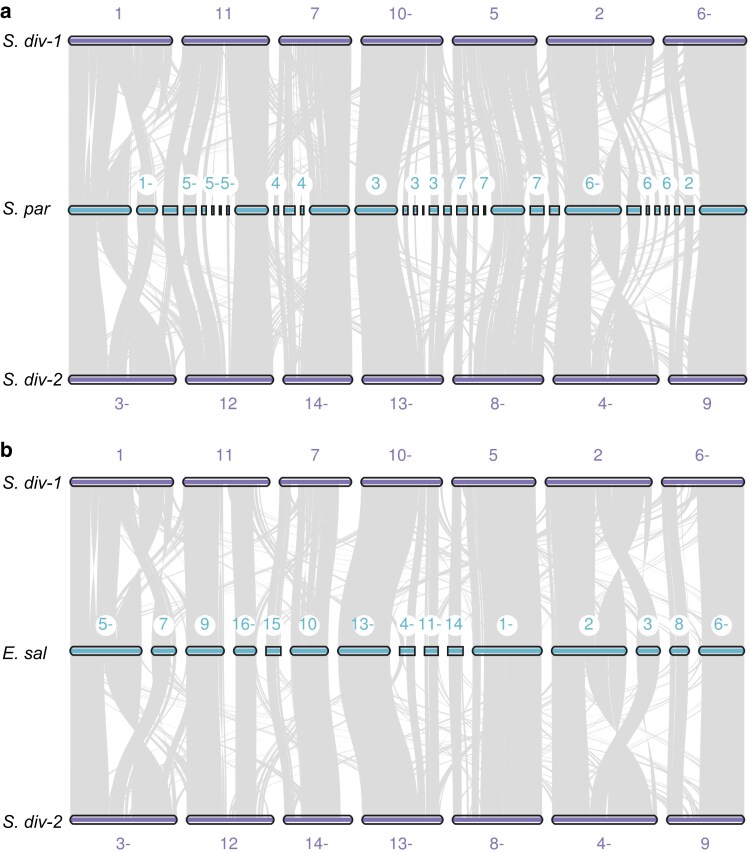
Synteny plots with *S. parvula* (A) and *E. salsugineum* (B). Plots produced with JVCI MCscan (Tang *et al*. 2008, 2024). The *S. diversifolius* homoeologous chromosomes were separated into 2 groups (shown in the top and bottom rows of each plot) to better illustrate the duplication. A “–” after a scaffold or chromosome number indicates that it is plotted in reverse orientation for better visualization.

**Fig. 5.**
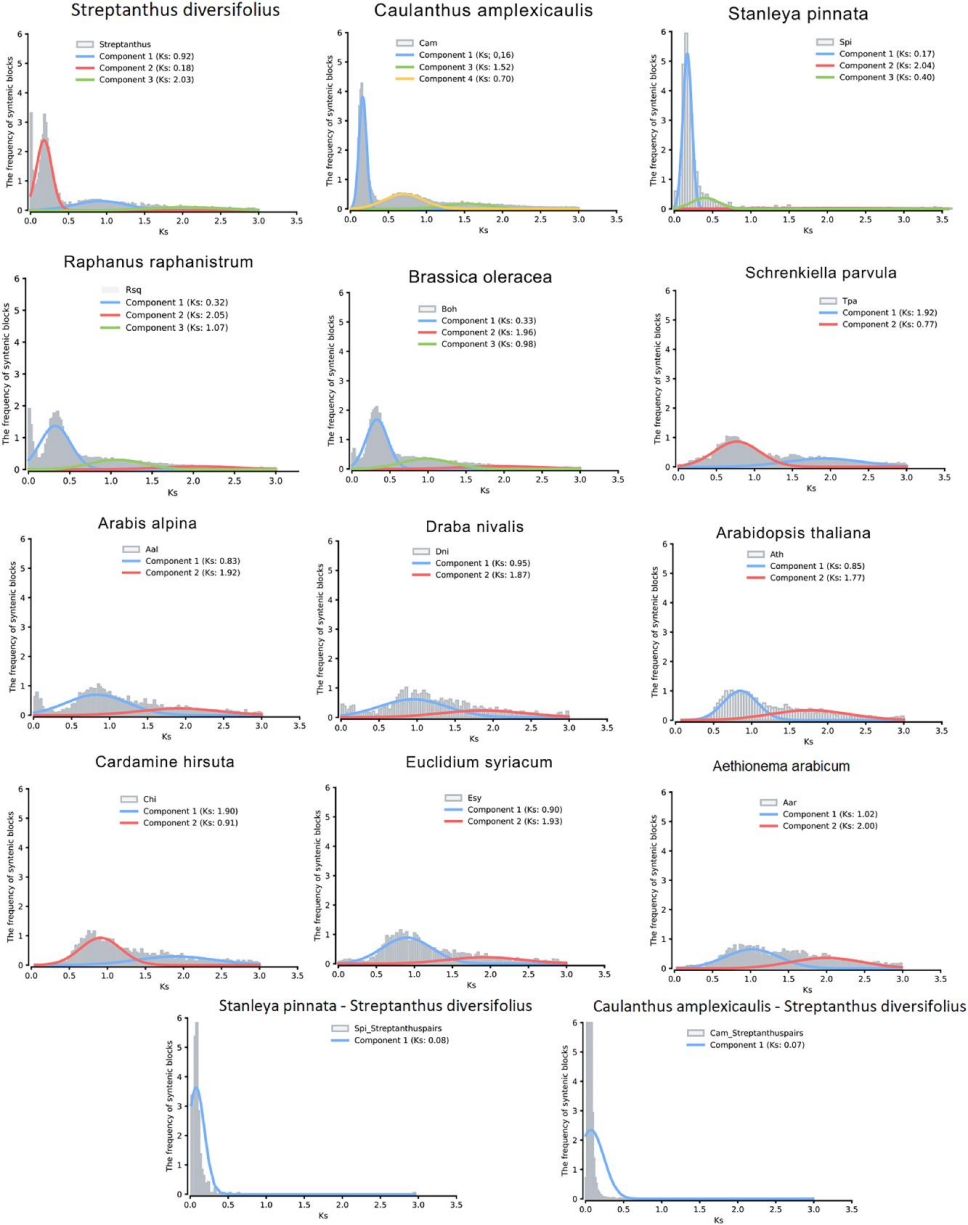
Top plots are intragenomic analysis of Ks for the analyzed species (excluding *T. arvense*, e*) salsugineum* and *A. lyrata*). Colored lines indicated distinct Ks peaks in each plot. Bottom plots are intergenomic analysis of Ks between *S. diversifolius* and either *S. pinnata* or *C. amplexicaulis*.

**Fig. 6.**
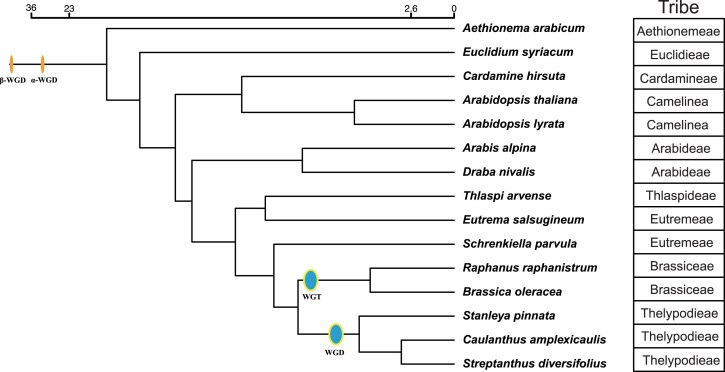
Phylogenetic species tree. Tree was inferred using phylogenetic trees from the 118 single-copy orthogroups identified between our 15 species of interest. Scale bar represents millions of years ago.

The genome alignment dot plot (Fig. 3a) provides additional evidence of a whole genome duplication shared among members of the Thelypodieae. To better visualize this duplication, we made alluvial synteny plots of *S. diversifolius* vs *A. thaliana* (Fig. 3b), and vs 2 species in the Brassicodae (II) but outside of the Thelypodieae (*S. parvula* and *E. salsugineum*; Fig. 4). In each comparison *S. diversifolius* shows a duplication relative to the comparison species. In contrast, a dotplot comparison of *S. diversifolius* with the Thelypodieae member *C amplexicaulis* illustrates that *C. amplexicaulis* shares the duplication (Supplementary Fig. 3 in file Supplemental Material; the *C. amplexicaulis* assembly is too fragmented for an alluvial plot). To investigate the whole genome duplication further, we examined the distribution of the synonymous substitution rate (Ks) among paralogs. In the absence of whole genome duplication events, the Ks distribution is expected to show a rapid decrease over time (Lynch and Conery 2000). Additional peaks in the Ks distribution indicate whole genome duplication events and can be used to estimate their age (Ohno 1970; Maere *et al*. 2005) Analysis of the Ks peaks for internal WGD gene pairs in *S. diversifolius* and the genomes listed in the “Genomic data sources” section revealed that *S. diversifolius* has a relatively new WGD with a Ks of ∼0.18, along with 2 older peaks at 0.92 and 2.03, likely corresponding to the Brassicaceae α WGD and the Eudicots γ WGT, respectively (Fig. 5). Our constructed phylogenetic tree (Fig. 6) agrees with other phylogenetic analyses (Cacho *et al*. 2014; Kagale *et al*. 2014; Mandáková *et al*. 2017) that indicate *S. diversifolius, C. amplexicaulis*, and *S. pinnata* are closely related, and both *C. amplexicaulis* and *S. pinnata* also exhibit a Ks peak around 0.18. No other species in our analysis shared the Ks peak around 0.18. To determine the species divergence time and ascertain if it is the same WGD we calculated the Ks values between *S. diversifolius* and *S. pinnata* and between *C. amplexicaulis* and *S. diversifolius*. These species show a divergence Ks peak of about 0.08, which is later than the WGD event. Combined with the synteny analysis, it appears that this WGD likely occurred around 7.4 MYA, and is shared by *S. diversifolius* and other members of the Thelypodieae.

**Fig. 7.**
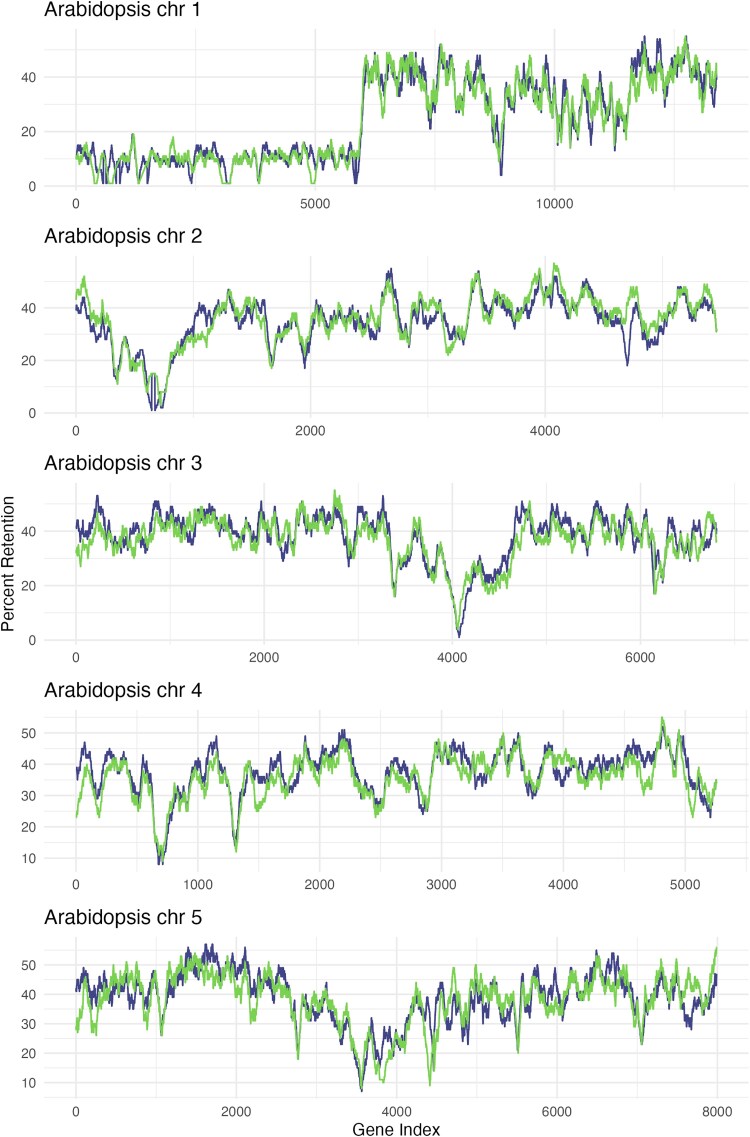
Fractionation bias test. For each 100-gene window in Arabidopsis, the percentage of genes retained on the 2 most homologous *S. diversifolious* scaffolds was calculated and plotted. The 2 colors represent the scores of the 2 homoeologs at each location. The lack of separation of the lines indicates no evidence of fractionation bias.

Whole genome duplication can result from non-disjunction during meiosis (creating an autotetraploid) or by hybridization of closely related species followed by genome doubling (creating an allotetraploid). We aimed to distinguish these 2 possibilities by comparing gene and repeat content of homoeologous chromosomes. It is well documented that genes are often retained unequally after allotetraploidization, with one sub-genome becoming more fractionated than the other (Thomas *et al*. 2006; Sankoff *et al*. 2010; Freeling *et al*. 2012). We performed a fractionation bias analysis using CoGe (Lyons and Freeling 2008; Joyce *et al*. 2017) to compare gene loss of homoeologous *Strepanthus diversifolius* scaffolds, relative to *Arabidopsis thaliana.* We found no evidence of fractionation bias (Fig. 7). For relatively recent allotetraploids, one also expects to find homoeolog-specific repeat sequences, because the repeat sequences will have diverged over evolutionary time in the 2 progenitors. To search for sub-genome specific repeats, we used SubPhaser (Jia *et al*. 2022) to look for kmers that could distinguish between homeologs. Of 4,425,565 kmers identified, only 88 were unique to one homeolog among a pair, and there was no significant enrichment of unique kmers on any homeolog. Taken together, these analyses suggest that the whole genome duplication described here either resulted from an autopolyploid event or from hybridization of 2 very closely related species. In a study of reproductive isolation in this clade, 40% of 40 crosses between *S. diversifolius* and closely related *S. tortuosus* set seed, indicating incomplete post-zygotic reproductive isolation, and possible opportunities for past and ongoing hybridization (Christie and Strauss 2018).

### Gene family expansion highlights ATP-related gene categories

**Fig. 8.**
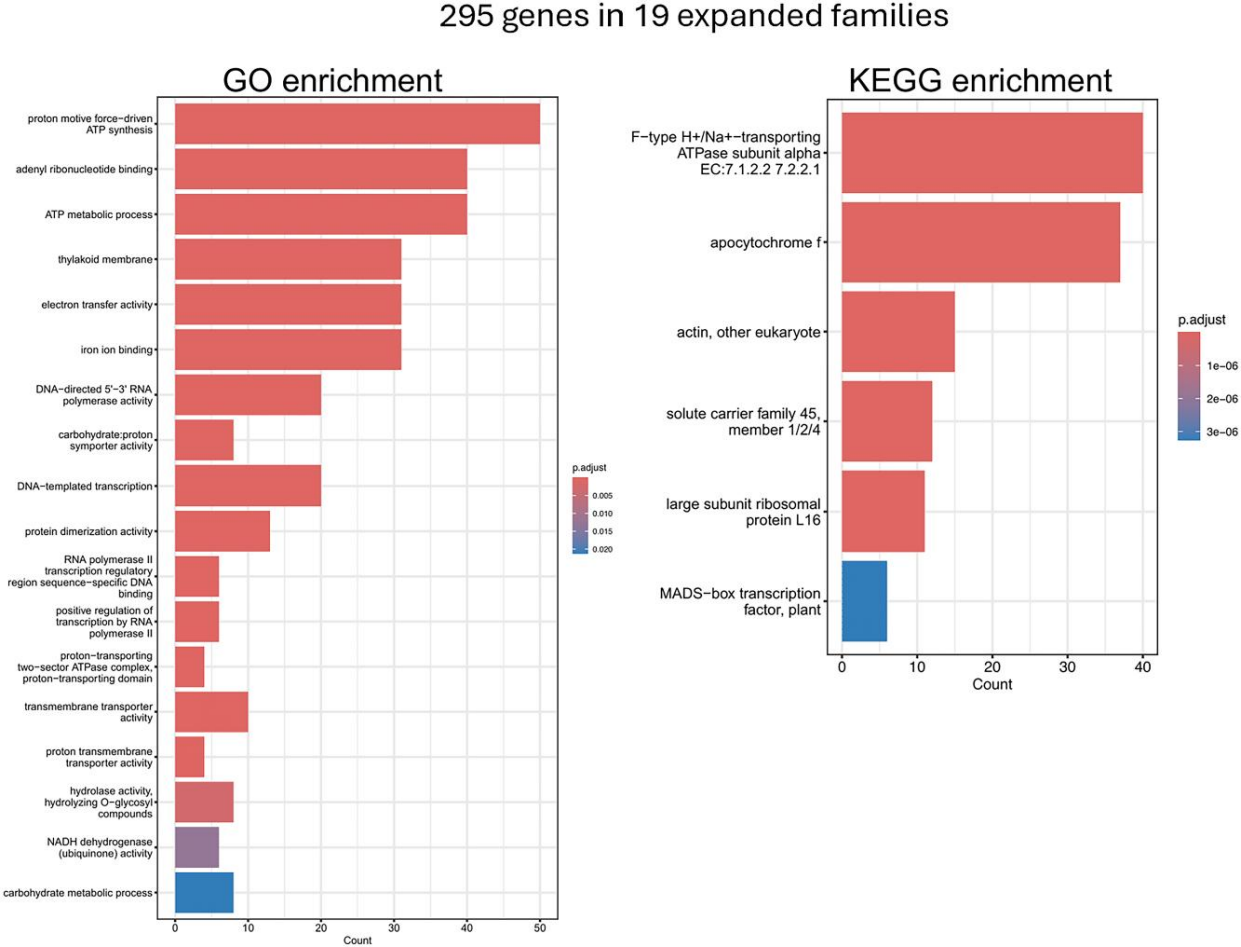
GO and KEGG enrichment analysis of the 295 genes in the 19 expanded gene families.

Confident in the presence of a WGD we further looked at the composition of the genome to assess if there were gene families that showed significant expansion. CAFE5 analysis reported a total of 19 expanded gene families containing a total of 295 genes (Fig. 8). GO and KEGG enrichment analysis were performed on this set of genes. GO analysis revealed an enrichment for several biological processes related to ATP. KEGG analysis also showed an enrichment for ATP-related processes with the largest most significant category being F-type H+/Na + -transporting ATPase subunit alpha-EC:7.1.2.2 7.2.2.1.

### Duplicate gene retention and loss

Following a WGD homoeologous genes may be retained as duplicates or revert to single copy, a process that is non-random and that varies by elapsed time and gene function (Thomas *et al*. 2006; Birchler and Veitia 2007; Freeling 2009; Duarte *et al*. 2010; De Smet *et al*. 2013; Conant *et al*. 2014; Li *et al*. 2016; Cheng *et al*. 2018). To determine if the patterns of gene retention and loss following the Streptanthoid WGD described here was consistent with previous observations in other species, we identified pangenes present in 2 outgroup species that do not have the WGD (*E. salugineium* and *S. parvula*) and in 2 Streptanthoid species that do have the WGD (*S. diversifolius* and *C. amplexicalus*). Genes were then categorized as those where duplicates were retained in both *S. diversifolius* and *C. amplexicalus*, those that reverted to single copy in both species, or those that were retained as duplicates in one species and single copy in the other. We then performed a GO over-representation analysis to determine if any categories of genes were enriched among the different categories of retention (Table 3). Among these results, we found that genes related to transcription (mostly transcription factors) were preferentially retained as duplicates in both species, whereas genes related to meiosis preferentially reverted to single copy in both species, consistent with prior results (Duarte *et al*. 2010; De Smet *et al*. 2013; Li *et al*. 2016; Cheng *et al*. 2018).

**Table 3.** Homoeolog retention and loss. Enriched gene ontology terms for different patterns of homeolog retention.

Category	S_div. #	C_amp. #	GO.ID	Term	Total Count	*n* in Group	Expected	*P*_val
both retained	2	2	GO:0006355	regulation of DNA-templated transcription	434	355	323.18	0.00024
both retained	2	2	GO:0006886	intracellular protein transport	91	80	67.76	0.00079
both single	1	1	GO:0005975	carbohydrate metabolic process	256	37	19.25	1.90E-05
both single	1	1	GO:0007140	male meiotic nuclear division	5	3	0.38	0.0056
both single	1	1	GO:0007143	female meiotic nuclear division	2	2	0.15	0.0056
both single	1	1	GO:0006508	proteolysis	188	21	14.14	0.0075
S_div. retained	2	1	GO:0009311	oligosaccharide metabolic process	18	4	0.98	0.0029
S_div. retained	2	1	GO:0055085	transmembrane transport	304	29	16.52	0.0042
C_amp. retained	1	2	GO:0009245	lipid A biosynthetic process	3	3	0.38	0.002
C_amp. retained	1	2	GO:0046493	lipid A metabolic process	3	3	0.38	0.002
C_amp. retained	1	2	GO:1901269	lipooligosaccharide metabolic process	3	3	0.38	0.002
C_amp. retained	1	2	GO:1901271	lipooligosaccharide biosynthetic process	3	3	0.38	0.002
C_amp. retained	1	2	GO:0006568	tryptophan metabolic process	6	4	0.75	0.003
C_amp. retained	1	2	GO:0006586	indolalkylamine metabolic process	6	4	0.75	0.003
C_amp. retained	1	2	GO:0008283	cell population proliferation	6	4	0.75	0.003
C_amp. retained	1	2	GO:0042430	indole-containing compound metabolic process	6	4	0.75	0.003
C_amp. retained	1	2	GO:0045132	meiotic chromosome segregation	4	3	0.5	0.0072
C_amp. retained	1	2	GO:0070192	chromosome organization involved in meiosis	4	3	0.5	0.0072
C_amp. retained	1	2	GO:0006576	biogenic amine metabolic process	11	5	1.38	0.0074
C_amp. retained	1	2	GO:0009451	RNA modification	99	21	12.45	0.0099

## Discussion

The Streptanthoid Complex is a collection of plants found throughout the CFP. Individuals in this collection can be found in environments ranging from arid deserts to alpine mountain tops (Baldwin *et al*. 2012). The *Streptanthus* (*s.l.)* clade overall has been identified as a group representing recent speciation (Christie and Strauss 2018) and about forty percent of its species are endemic to serpentine soils (Raven 1978; Safford *et al*. 2005; Cacho and Strauss 2014). Despite living in drastically different environments, these flowering plants share a highly connected genetic background (Christie and Strauss 2018). Phylogenetically close relationships along with the expansive range and diverse morphology make this collection of plants an ideal study subject for exploring the diversification and adaptation of flowering plants in the CFP. Active research is currently being conducted on this complex (Cacho *et al*. 2014, 2015, 2021; Cacho and Strauss 2014; Weber *et al*. 2018; Gremer *et al*. 2020; Worthy *et al*. 2024) but is restricted to variation in traits due to a lack of genomic resources. This telomere-to-telomere assembly offers a starting point in expanding the realm of genomic analyses related to these species. Possible uses of this assembly include comparative analyses that examine genome rearrangements and gene copy number variation in species adapted to different environments; serving as a reference for mapping RNA-sequencing reads to identify gene expression differences (and therefore candidate mechanisms of adaptation) in populations or species native to different environments; genome-wide association and quantitative trait locus mapping studies to connect genotype to phenotype; and a reference for population or phylogenetic studies examining targets of positive selection.

The assembly presented here provides further evidence of a recent whole genome duplication shared throughout the Thelypodieae that contains the Streptanthoid Complex. Whole genome duplications have previously been described in other members of the tribe, including *S. farnsworthianus* (Mandáková *et al*. 2017), *C. amplexicaulis* (Burrell *et al*. 2011), *P. antiscorbutica* and *Stanleya pinnata* (Kagale *et al*. 2014). Prior studies used transcriptome or molecular marker analysis. We build on these studies by providing a whole genome assembly that can be used to study genome evolution after the whole genome duplication, through our synteny analysis that shows the structure of the duplicated chromosomes, our analysis indicating that the WGD likely resulted from an autopolyploid event, and through our cross-species Ks analysis that indicates there was a single, shared WGD event among these species.

Gene duplication, such as from the WGD described here, is a critical component of evolution, allowing rapid evolution of new functions (Ohno 1970). Whole genome duplications can be due to allo- or autotetraploidy. Historically it was thought that allotetraploids were more common than autotetraploids (Clausen *et al*. 1940; Stebbins 1947), however more recent analysis shows that auto- and allotetraploids exist in roughly equal proportions among plant species (Barker *et al*. 2016). Allotetraploidy can provide an immediate advantage due to combining divergent alleles that can broaden the organism's environmental niche and stress tolerance (Brochmann *et al*. 2004; Doyle *et al*. 2008; te Beest *et al*. 2012; Van de Peer *et al*. 2021), but autotetraploidy such as is seen here, can still enable rapid evolution of new functions (Ohno 1970; Parisod *et al*. 2010; Ebadi *et al*. 2023). Following a whole genome duplication event, there can be an extensive process of genome rearrangement and consolidation as diploidization occurs (Lynch and Conery 2000; Qiao *et al*. 2019). During this time, genes and their functions can be lost, gained, and changed through processes including subfunctionalization and neofunctionalization (Lynch and Conery 2000; Qiao *et al*. 2019; Almeida-Silva and Van de Peer 2023). It has been reported that the whole genome duplication event shared among the order Brassicales expanded the ability of plants in this order to produce glucosinolates (Barco and Clay 2019).

It is possible that the recent whole genome duplication described here played a role in diversification of the Streptanthoid Complex throughout California. *Streptanthus* species may have been able to rapidly adapt to diverse and harsh climates and environments such as serpentine soils through the increased genetic arsenal following the whole genome duplication (Ohno 1970; Qiao *et al*. 2019). Across the clade, soil environments are highly variable, especially with respect to Ca:Mg ratios (Cacho and Strauss 2014), suggesting the presence of mechanisms able to regulate relative uptake of these elements. For example, the enrichment of ATP-related terms among expanded gene families is of interest. While *S. diversifolius* is not found on serpentine soil, field soil analyses from the DNA collection site at Table Mountain revealed that both Ca^2+^ and Mg^2+^ levels are especially low (0.27 and 0.1 meq/100 g, respectively) relative to soils of other species in the clade (Cacho and Strauss 2014, Supplementary Suppl. data); any soil less than 0.5 meq/100 g Mg^2+^ is considered low in Mg (Horneck *et al*. 2023). Furthermore, Ca:Mg at the site (2.7) is lower than the vast majority of non-serpentine soils in the clade (range 0.48–85), as well as that of Hoaglands' solution (4.0), suggesting relative Mg enrichment. Chelation of adenylates by magnesium (Mg) is an essential feature of cell metabolism (Kleczkowski and Igamberdiev 2021), therefore the enrichment in ATP-related terms could be related to ATP homeostasis in soil with overall low [Mg^2+^] or low Ca^2+^:Mg2^2+^ ratio. Enrichment of ATP terms could also be related to the overall low [Ca^2+^], given the importance of ATP and Ca^2+^ ATPases in Ca^2+^ transport and homeostasis (Schumaker and Sze 1985; Tang and Luan 2017). ATP-related genes and terms have been found in other studies of soil adaptation, for example, The GO term “ATPase activity” was identified as enriched in a genomic comparison of serpentine and granitic populations of *Arabidopsis lyrata* (Turner *et al*. 2008) and a heavy metal ATPase was identified as underlying a Cd tolerance QTL in *Arabidopsis halleri* (Courbot *et al*. 2007). Other studies have identified Ca, Mg, and metal ion transporters and signaling as being associated with or important for soil adaptation (Bradshaw Jr 2005; Turner *et al*. 2010; Arnold *et al*. 2016).

Another interesting aspect of whole genome duplications is that the likelihood for duplicated genes to be lost or retained varies for genes in different functional categories and across evolutionary time (Freeling 2009; Duarte *et al*. 2010; De Smet *et al*. 2013; Conant *et al*. 2014; Li *et al*. 2016; Cheng *et al*. 2018). There are many mechanisms that have been proposed to impact biased retention or loss (reviewed by Cheng *et al*. 2018); within the time-frame of the WGD described here, retention to avoid dosage imbalance (Birchler and Veitia 2007; Freeling 2009) and subfunctionalization (Force *et al*. 1999; Lynch and Force 2000) are likely important mechanisms favoring retention (Cheng *et al*. 2018). Among genes retained as duplicates in both *S. diversifolius* and *C. amplexicaulis,* we found an enrichment of genes involved in transcription, a common finding that may be due to the importance of dosage balance and/or subfunctionalization. We also found enrichment for genes involved in membrane transport, which is consistent with retention due to the gene balance hypothesis. Genes that quickly revert to single copy tend to be conserved “housekeeping” genes and/or those that function on their own instead of in connected networks or complexes (Duarte *et al*. 2010; De Smet *et al*. 2013; Cheng *et al*. 2018). Among genes that reverted to single copy in both species, we found an enrichment of genes with the GO term “Proteolysis”. Although this term has not previously been found to be under-represented among retained duplicates nor over-represented among single-copy genes (Duarte *et al*. 2010; De Smet *et al*. 2013; Li *et al*. 2016), genes in this category may be working relatively independently or in a housekeeping function. De Smet *et al* (2013) found that the GO term “Meiosis I” was overrepresented among single-copy plant genes, consistent with our identification of 2 terms related to meiosis among genes that reverted to single copy in both species. We also found over-representation of the GO term “carbohydrate metabolic process” in genes that reverted to single copy in both species; this is different from the general pattern reported by (Li *et al*. 2016), who found that genes in this category are most often retained as multi-copy over long evolutionary time frames. Lastly, genes retained as duplicates in one of the 2 species may result from different selective pressures favoring retention and neofunctionalization of different processes in the 2 species, or could reflect stochastic differences, or may be due to differences in annotation. Perhaps most interesting in this category is that we find that 3 of 3 genes tagged with terms related to lipid A and lipooligosaccharides are retained as duplicates in *C. amplexicaulis* but not in *S. diversifolius.* While plants do not make lipid A, they do have a related biosynthetic pathway (Li *et al*. 2011), expression of genes in this pathway are stress responsive in rice (Luo *et al*. 2019), and knockdown of rice *LPXC,* a homolog of one of the genes retained in duplicate in *C. amplexicaulis,* causes reduced tolerance to cold stress (Islam *et al*. 2023). Thus, it is possible that differential retention of genes in the Lipid A pathway is related to different stresses faced by *C. amplexicaulis* and *S. diversifolius.*

Future research should be directed towards identifying genes that allowed *Streptanthus* to expand its geographic and climatic range. Identification of these genes could have major impacts on the agricultural field given the close genetic relationship between *Streptanthus* and it economically important sister clade *Brassica.* More generally, identifying these genes will improve our understanding of the mechanisms by which plants can evolve adaptations to different environments. The telomere-to-telomere assembly described here is a key resource that will enable future genetic studies to identify critical environmental adaptation genes in Arabidopsis.

## Supplementary Material

jkaf022_Supplementary_Data

## Data Availability

Genome sequencing data and assembly data can be found on NCBI under Bioproject PRJNA283414. Transcriptome data used for annotation can be found on Dryad at https://doi.org/10.5061/dryad.t1g1jwt99 and on NCBI under Bioproject PRJNA992064. Annotations, associated scripts, and plotting code can be found on Github at https://github.com/MaloofLab/Davis_Dimensions_Sdiv_Assembly. Download information for sequencing data used in this project is described in Methods/Genomic data sources. Supplemental material available at G3 online.
