# Supplementary material for: A chromosome-level genome assembly of the varied leaved jewelflower, Streptanthus diversifolius, reveals a recent whole genome duplication
Source: G3 (Bethesda). 2025 Mar 18;15(4):jkaf022. doi: 10.1093/g3journal/jkaf022 (PMC12005169; doi:10.1093/g3journal/jkaf022)
Supplement: jkaf022_Supplementary_Data [file jkaf022_supplementary_data.pdf]

Supplemental Figures:

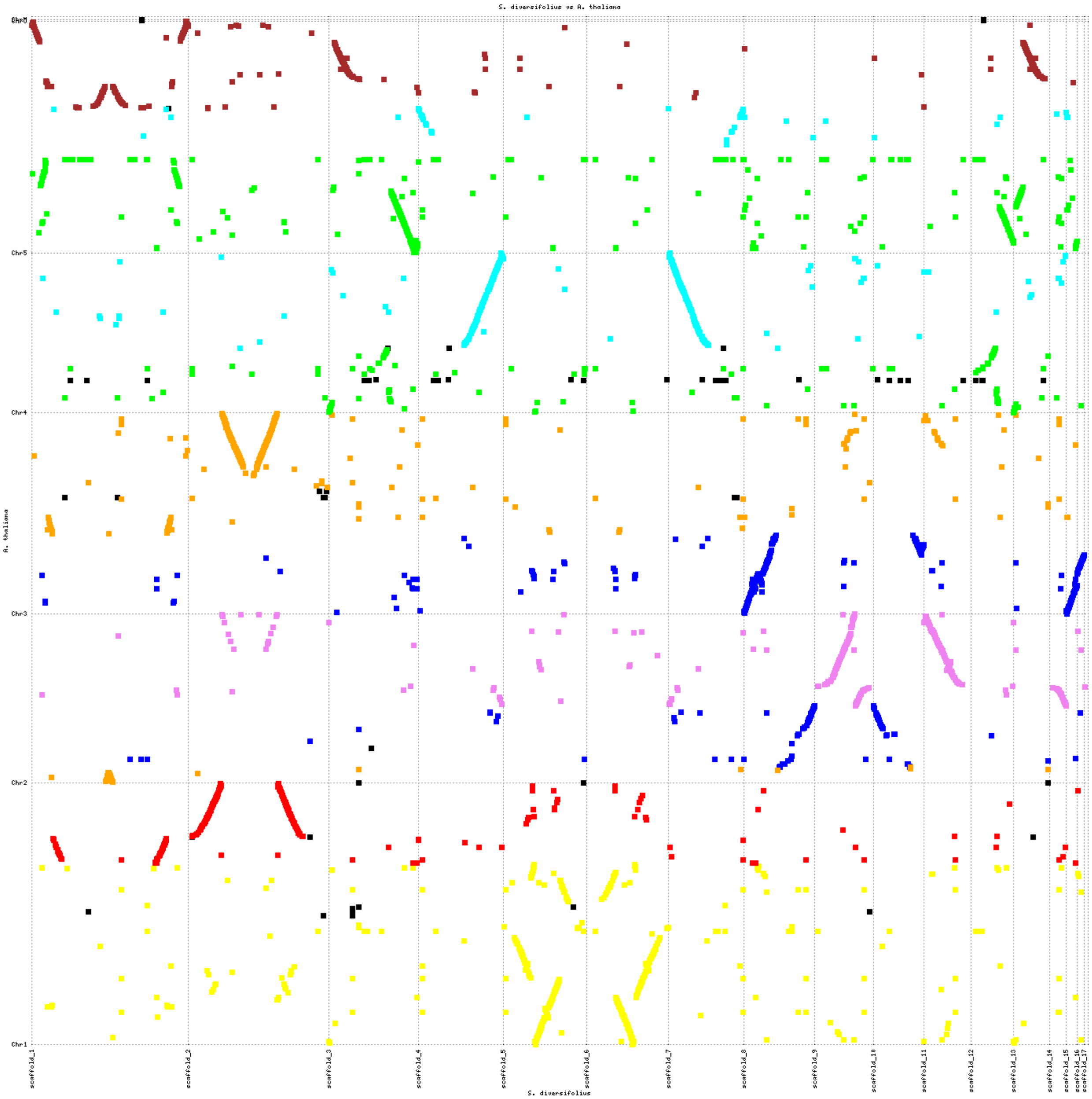

Figure S1. All scaffolds greater than 1 Mb in the HiC assembly aligned to the TAIR 10 A. thaliana genome assembly. Alignment was performed using Promer and plotted using mummerplot, both programs included in the MUMmer bioinformatic toolkit. The color of each line corresponds to the 8 ancestral crucifer karyotype (ACK) blocks of A. thaliana apart from black which corresponds to regions which do not belong to an ACK block. ACK block boundaries are based on gene locations summarized in Lysak et. Al 2016. Gray dashed lines indicate separate scaffold in each assembly.

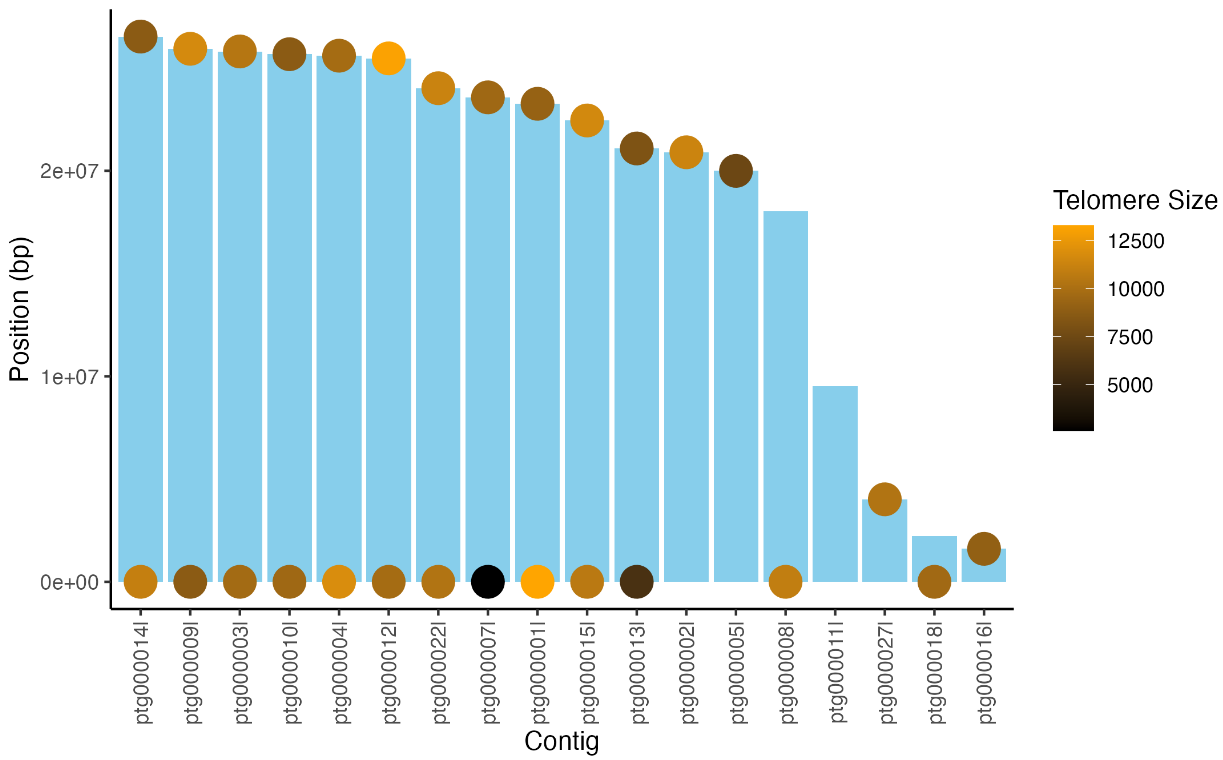

Figure S2 Illustration of telomere repeat location on the 18 scaffolds larger than 1 Mb.

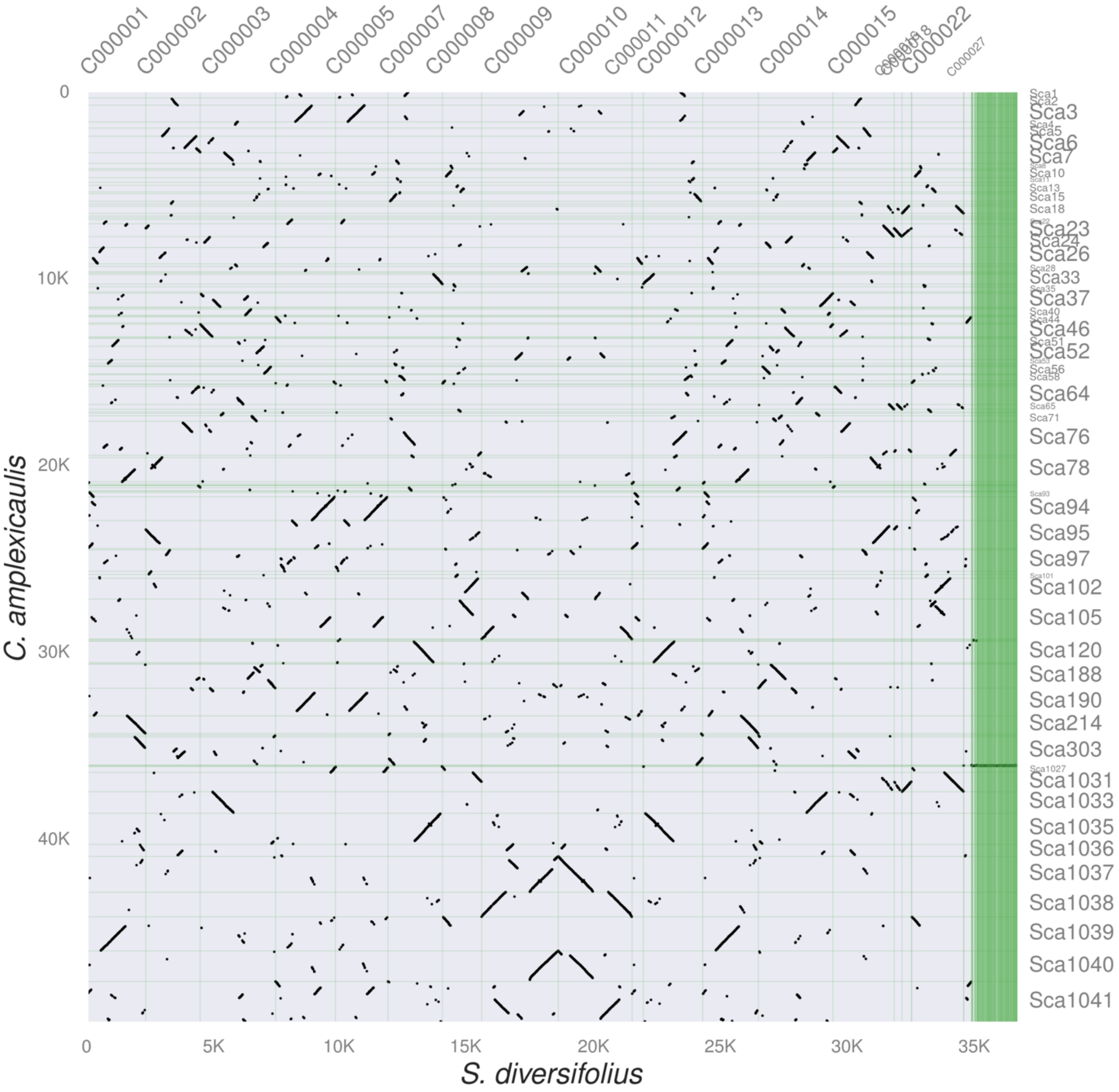

Figure S3. Dot plot comparison of *C. amplexicaulis* and *S. diversifolius.* Looking across rows or up columns most of the larger scaffolds show two regions of homology indicating that both species have a genome duplication.

Supplemental Tables

Table S1 Number of genes, isoforms, and BUSCO summary statistics for each transcriptome. Analysis completed using BUSCO version 5.5.0 in transcriptome mode and the embryophyta odb10 dataset

Species	Genes	Isoforms	Complete BUSCOs	Single Copy	Duplicated	Fragmented	Missing	Total
C. amplexicaulis	22,629	84,786	91.9%	15.9%	76.0%	1.9%	6.2%	1614
C. anceps	21,822	90,802	91.8%	16.7%	75.1%	1.7%	6.5%	1614
C. inflatus	21,459	79,422	94.3%	22.4%	71.9%	1.2%	4.5%	1614
S. breweri	22,475	65,787	94.7%	25.9%	68.8%	1.4%	3.9%	1614
S. glandulosus	21,612	79,150	93.4%	19.2%	74.2%	1.7%	4.9%	1614
S. tortuosus	21,621	75,657	94.3%	21.6%	72.7%	1.7%	4.0%	1614

Table S2 Summary of gene annotation

Measurement	Value
Number of genes	40,605
Number of mRNAs with UTR both sides	14172
Number gene overlapping	2949
Number of single exon gene	8430
Mean exons per mRNA	5.7
Mean mRNA length	1377
Longest mRNA	16,449
Shortest mRNA	22

Table S3 Repeat element content of HiFi assembly. Analysis completed using RepeatMasker version 4.1.5 in sensitive mode. Query species was assumed to be Brassicales.

			Number of elements	Length occupied (bp)	percentage of sequence (%)
**Retroelements**			74,066	75,569,968	18.79
	SINEs:		270	39,770	0.01
	Penelope:		0	0	0
	LINEs:		4,782	3,064,392	0.76
		CRE/SLACS	0	0	0
		L2/CR1/Rex	0	0	0
		R1/LOA/Jockey	0	0	0
		R2/R4/NeSL	0	0	0

		RTE/Bov-B	0	0	0
		L1/CIN4	4,758	3,061,849	0.76
	LTR elements		69,014	72,465,806	18.02
		BEL/Pao	0	0	0
		Ty1/Copia	32,174	42,611,770	10.6
		Gypsy/DIRS1	33,841	28,849,738	7.17
**DNA transposons**			20,796	6,705,897	1.67
	hobo-Activator		4,574	1,292,104	0.32
	Tc1-IS630-Pogo		1,476	311,487	0.08
	En-Spm		0	0	0
	MULE-MuDR		6,750	2,499,600	0.62
	PiggyBac		0	0	0
	Tourist/Harbinger		1,260	438,400	0.11
	Other (Mirage, P-element, Transib)		0	0	0
**Rolling-circles**			994	159,073	0.04
**Unclassified:**			23	4,153	0
**Total interspersed repeats:**				82,280,018	20.46
**Small RNA**			7,919	12,239,420	3.04
**Satellites:**			58	10,415	0
**Simple repeats:**			120,687	16,567,216	4.12
**Low complexity:**			26,984	1,340,333	0.33
